# Forecasting Unplanned Purchase Behavior under Buy-One Get-One-Free Promotions Using Functional Near-Infrared Spectroscopy

**DOI:** 10.1155/2022/1034983

**Published:** 2022-11-07

**Authors:** SuJin Bak, Minsun Yeu, Jichai Jeong

**Affiliations:** ^1^Department of Brain and Cognitive Engineering, Korea University, Seoul 02841, Republic of Korea; ^2^College of Business Administration, University of Ulsan, Ulsan 44610, Republic of Korea

## Abstract

It is very important for consumers to recognize their wrong shopping habits such as unplanned purchase behavior (UPB). The traditional methods used for measuring the UPB in qualitative and quantitative studies have some drawbacks because of human perception and memory. We proposed a UPB identification methodology applied with the brain-computer interface technique using a support vector machine (SVM) along with a functional near-infrared spectroscopy (fNIRS). Hemodynamic signals and behavioral data were collected from 33 subjects by performing Task 1 which included the Buy-One-Get-One-Free (BOGOF) and Task 2 which excluded the BOGOF condition. The acquired data were calculated with 6 time-domain features and then classified them using SVM with 10-cross validations. Thereafter, we evaluated whether the results were reliable using the area under the receiver operating characteristic curve (AUC). As a result, we achieved average accuracy greater than 94%, which is reliable because of the AUC values above 0.97. We found that the UPB brain activity was more relevant to Task 1 with the BOGOF condition than with Task 2 in the prefrontal cortex. UPBs were sufficiently derived from self-reported measurement, indicating that the subjects perceived increased impulsivity in the BOGOF condition. Therefore, this study improves the detection and understanding of UPB as a path for a computer-aided detection perspective for rating the severity of UPBs.

## 1. Introduction

Consumers have experienced financial problems such as excessive consumption, household debt, and monetary losses because of unplanned purchases [1]. Many studies have shown that reasonable consumption is difficult because unplanned purchase behavior (UPB) occurs emotionally or impulsively [2]. Many studies have reported that UPB can occur under situations that encourage people's impulsiveness such as price discounts and time pressures [3, 4]. UPBs are defined as a purchase of any item that consumers had not planned to purchase before entering the shops [5]. UPBs are increased by promotion strategies [6] such as price discounts, coupons, and money-back guarantee. Especially, “Buy-One-Get-One-Free” (BOGOF) is one of the most popular promotion strategies. A previous study found that over 53.3% of 192 respondents preferred BOGOF over other promotions [7]. This promotion strategy plays an important role in eliciting consumer's UPBs.

**Figure 1:**
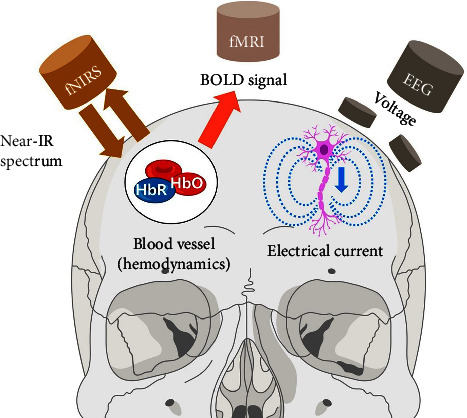
Noninvasive mapping of brain function using neuroimaging technologies. EEG records voltage fluctuations caused by electrical currents flowing through the brain because of active neurons using an array of electrodes placed on the scalp. fMRI measures hemodynamic responses associated with brain activity by relying primarily on the local blood-oxygen-level-dependent (BOLD) signal, which detects changes in blood oxygenation caused by neural activity. fNIRS measures the changes in oxygenation and deoxygenation hemoglobin concentrations in the brain using near-infrared light.

To assess whether consumers' UPB is, there are traditional research methods such as interviews, surveys, and questionnaires [8]. However, they rely on consumers' subjective perceptions and memories [9]. Furthermore, there is still a lack of tools and equipment for empirically measuring UPBs. To solve this issue, there are recent studies that have reported empirical evidence for unplanned purchases through brain signal measuring equipment [10]. There are noninvasive equipment for brain measurements such as electroencephalogram (EEG) [11, 12] and functional magnetic resonance imaging (fMRI) [13].  Figure 1 describes the noninvasive brain signal measurement equipment that is harmless to the human body. EEG records voltage fluctuations caused by electrical currents flowing through the brain cortex because of neural activity [14]. fMRI uses the blood-oxygen-level-dependent (BOLD) contrast to detect changes in blood oxygenation that occur in response to neural activity, and it has become the most common method for imaging brain functions in vivo [15]. However, fMRI is unsuitable for certain research applications and various clinical applications because fMRI is physically prone to mixing motion artifacts, exposes to loud noises, and is expensive. To compensate for the shortcomings of fMRI, fNIRS has become a promising imaging modality for UPB evaluation as well as for reducing the physical space constraint and costs of fMRIs [16, 17]. fNIRS is one of the state-of-the-art brain signal measurement equipment, especially with scalability, convenience in use, and portability [18, 19]. With the benefits of the fNIRS, we can provide valuable insights into the consumers' UPB by using the brain-computer interface (BCI) technique [20]. We utilize the BCI technique to explain customer behaviors in detail [21], and it will benefit to neuromarketing industries using fNIRS utilities [22].

**Figure 2:**
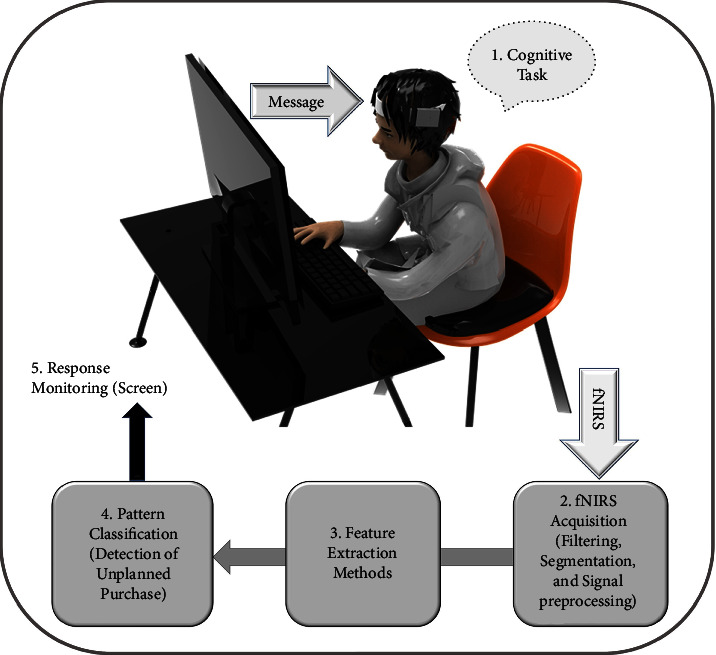
Typical scheme of a BCI system consisting of five main stages. The process is as follows: (1) cognitive task; (2) fNIRS acquisition; (3) feature extraction methods; (4) pattern classification; (5) response monitoring. We went through this whole process to detect UPB.

A general scheme of BCI can be explained using five main steps, as illustrated in Figure 2. In Step 1, the people should perform cognitive tasks with (or without) the BOGOF condition. The brain signal changes according to the cognitive tasks and these brain signals are acquired by an fNIRS device and then gets transmitted to the next step. In Step 2, the fNIRS signals are digitized, amplified, and filtered to delete undesired signals called artifacts such as physiological noise. Then, the clean signals go to Step 3, where the clean signals extract features to be used as a descriptor of the fNIRS signals for classifying the UPB patterns. The features are classified into UPB and non-UPB states in Step 4. Finally, the message which indicates the UPB classification result is presented on a computer screen in Step 5. Through these processes, this study shows that the UPB and non-UPB can be distinguished because of brain signals that reflect people's actual cognitive consequences.

Accordingly, we proposed a methodology to measure the UPB by the BCI technique. For this purpose, we acquired fNIRS signals during the cognitive tasks with and without BOGOF at online shopping shops and then converted them into preprocessed feature vectors by six time-domain feature extraction methods. To classify UPBs and non-UPBs, we adopted SVM, which is a widely used supervised learning approach with 10-fold cross-validation. We also used the “area under the receiver operating characteristic (AUC),” a measurement method for determining whether the results of SVM classifying UPB and non-UPB were reliable. As a result, we achieved an average accuracy of above 94% for classifying UPBs across all subjects, which also ensured the reliability of the results by obtaining an AUC value above 0.97. We observed that low brain activities were exhibited during Task 1 which included the BOGOF condition, and high brain activities were exhibited during Task 2 which excluded the BOGOF condition at the PFC. It is interpreted that there is a clear difference in the fNIRS signals depending on the BOGOF conditions. Furthermore, our experimental tasks are well designed because self-reported results indicate that these experimental tasks sufficiently induced the consumers' impulsiveness. Therefore, we believe that this study can be applied to a variety of applications by improving the accuracy of detecting UPB patterns under BOGOF conditions.

## 2. Materials and Methods

### 2.1. Subject

The study was approved by the Korea University Institutional Review Board (KUIRB-2022-0126–01) and then written informed consent was obtained from all subjects. Considering possible dropouts, we recruited 38 healthy adults but 5 people were excluded because of insufficient signal quality, and the remaining 33 subjects (mean ± standard deviation aged 24 ± 2.64 years) completed the entire study. There were 12 males (aged 24 ± 1.75 years) and 21 females (aged 24 ± 3.03 years) with normal or corrected to normal eyesight. All subjects were right-handed to minimize variability in brain signals. The subjects had no previous history of physical, mental, or psychological disabilities. All subjects were asked to minimize head movements and actively take part in the experiment as much as possible.

### 2.2. Experimental Procedures

**Figure 3:**
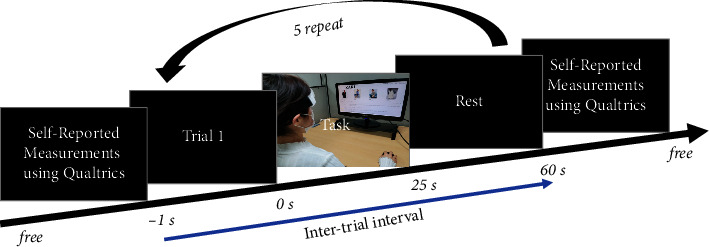
Overall procedures of performing Tasks 1 and 2. Participants are free to choose what clothes they want to buy from online shopping malls, which are divided into Task 1 with BOGOF conditions and Task 2 without BOGOF conditions. The whole experiment consists of trial number mark, experimental task, and rest. Each task is conducted for 25 s followed by a rest for 30 s. A total of five trials were conducted. The tasks are randomized and counterbalanced in sequence. Before and after the experiment, a self-reported measurement related to UPB was conducted using the popular survey platform, Qualtrics.

To investigate brain activation patterns, we designed two experimental tasks depending on the presence or absence of BOGOF.  Figure 3 illustrates the overall experiment protocol. Each experimental task comprised 5 trials where the subjects were asked to decide on purchasing displayed products under Task 1 (including BOGOF condition) and Task 2 (excluding BOGOF condition). In each task, the participants are free to choose the clothes they want [23]. The selected clothes brand is ZARA [24], known as the global specialty retailer of private label apparel fashion brand, which was selected by a Google survey in Koreapas [25], which is one of the major online communities for Korean university students. The clothing lines are divided into 5 major categories, which were displayed on the screen in the following order: knitwear, coat, vest, pants, and suit. There were 4 products in each group. For example, the coat group has four products (i.e., wool coat, classic long trench coat, wool mannish coat, and checked coat). The subjects can freely purchase up to 4 products they want in each group; in other words, they can purchase products from 0 to 20 per task. Each product was presented only once during the experiment. Each task lasted for 5 minutes and included the following stages (trial number display (1 s), task (25 s), rest (30 s)), and a brief buzzer (58.4 dBA, sound level meter, YATO, China). The order of the tasks was randomized and counterbalanced. All the subjects completed a self-reported measurement using Qualtrics, which is a popular survey platform, before and after the experiment.

### 2.3. fNIRS Equipment

**Figure 4:**
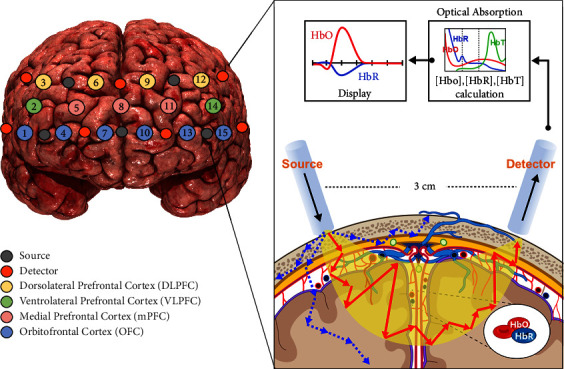
fNIRS channel configuration (a) and source-detector pair (b). In the left panel, the grey circles indicate the sources, whereas the orange circles represent the detectors, resulting in a total of 15 fNIRS channels in the prefrontal cortex (PFC). According to the 10–20 international system, the probe sets are symmetrically placed at FPz between Chs. 7 and 10. On the right panel, the source-detector pair measures lights from a diffuse volume of tissue beneath the pair as shown in the model of light propagation. These lights can reach approximately 8 mm into the brain cortex at a source-detector spacing of 3 cm. Lights at two wavelengths (780 nm and 850 nm) are used to reconstruct changes in oxy- and deoxy-hemoglobin concentrations from the modified Beer–Lambert law. A detector captures the lights resulting from the interaction with HbO and HbR, following a crescent-shaped path back to the surface of the skin. The crescent-shaped paths depict the traveling area of the near-infrared light (NIR) photons, while the blue dotted arrows indicate the light scattering. The red-colored arrows show the extra distance traveled by photons, which is corrected by the differential path length factor.

To measure the brain's hemodynamic responses in the prefrontal cortex (PFC), we used an fNIRS device (NIRSIT Lite, OBELAB Inc., Korea). To provide detailed guidance on the specifications of fNIRS, Figure 4 illustrates the fNIRS channel configuration covering the forehead and an example of source-detector pairs in detail. On the left panel, the fNIRS device has a total of 15 fNIRS channels composed of 5 sources (the grey circles) and 7 detectors (the orange circles). The probe sets are symmetrically arranged at FPz between Chs. 7 and 10 according to the 10–20 international systems. They were divided into 4 regions: the Dorsolateral prefrontal cortex (DLPFC), Ventrolateral prefrontal cortex (VLPFC), Medial prefrontal cortex (mPFC), and Orbitofrontal cortex (OFC). In these four areas, brain activations are known to primarily inhibit impulsivity [26]. Among them, VLPFC has separate left and right functions, and the left VLPFC is related to the reward system [27]. These results can help interpret hemodynamic activation patterns in the PFC that occur when performing our experimental tasks. On the right panel, the detector measures the lights from a diffuse volume of tissue in accordance with the model of light propagation. These lights can reach 8 mm into the brain cortex while maintaining a distance of 3 cm between the source and detector. The fNIRS device reflects the absorption properties of living tissues to measure changes in the local concentrations of oxy-hemoglobin (HbO) and deoxy-hemoglobin (HbR) within the crescent-shaped near-infrared region through the skull [28, 29]. The crescent-shaped paths represent the near-infrared light (NIR) photons' traveling area, while the blue dotted arrows represent light scattering. The red-colored arrows show the distance traveled by photons, which is corrected by the differential path length factor. Consequently, fNIRS can measure the hemodynamic changes quantitatively by absorbing near-infrared rays into the scalp and by measuring the emitted light emitted.

Based on the aforementioned principles of fNIRS, we recorded the optical density data at a frequency of 8.138 Hz and configured it to detect hemodynamic activity at wavelengths of 780 nm and 850 nm. The optical density data were bandpass filtered digitally in the range of 0.01–0.1 Hz to eliminate possible physiological signals such as respiration, heart rate, and unwanted noise. Filtered signals were converted to oxygenated and deoxygenated concentration changes using the modified Beer–Lambert law [30], and then the data were segmented into epochs ranging from −1 to 60 s relative to the task onset (0 s). The epoch was subjected to a baseline correction to subtract the mean value within a reference interval from −1 to 0 s. The temporal means of the fNIRS data in each channel were calculated by averaging the fNIRS data from the start to the end time (0–60 s) in each epoch. In this study, we handle only HbO signals because they have a higher signal-to-noise ratio [31, 32]. It means that the signal strength is stronger than the noise intensity. HbO is also regarded as a more reliable indicator for analyzing the PFC activation [33]. The acquired fNIRS dataset, as well as all related information, can be downloaded from https://github.com/SujinBak/BOGOF.

### 2.4. Extraction of Six Time-Domain Features: Mean, Variance, Kurtosis, Skewness, Slope, and Area

Feature extraction is an important step in extracting and maximizing the information that describes the unique property of the fNIRS signals. This step forms the features extracted from the brain signals into vectors. These feature vectors are recognized by the classifier, which makes it easier to classify two or more classes. The widely used feature extraction methods were divided into three main categories: time-domain analysis; frequency-domain analysis; and time-frequency domain analysis. We focused on time-domain analysis, which facilitates the understanding of the transient characteristics of physiological signals, including fNIRS signals [34]. It has been reported that time-domain features can improve the classification accuracy between different cognitive states [35]. Especially, the time-domain features represent the property difference between the measured signals, which is visually recognizable when an unexpected abnormality appears in the signals [36]. Accordingly, we adopted the framework for feature extraction used by Park and Dong. [37] and then calculated 6 time-domain features (mean, variance, kurtosis, skewness, slop, and area) to extract information across data [38]. We denote mean, variance, kurtosis, skewness, slope, and area as SM, SV, KR, SK, SS, and SA, respectively. Signal mean (SM) can be calculated by the following equation:

(1) $$\begin{array}{c} SM=\frac{1}{N}\overset{N-1}{\underset{n=0}{\sum }}x\left[n\right]\text{,} \end{array}$$

where *x*[*n*] is the input signal (∆HbO) at the time index of *n*, and *N* is the total length of the signals. Signal variance (SV) is calculated as follows:

(2) $$\begin{array}{c} SV=\frac{1}{N-1}\overset{N-1}{\underset{n=0}{\sum }}{\left(x\left[n\right]-\mu \right)}^{2}\text{,} \end{array}$$

where *μ*(=SM) is the mean found from (1). For signal kurtosis (KR), it is calculated by the following equation:

(3) $$\begin{array}{c} KR=E\left[{\left(\frac{x\left[n\right]-\mu }{\sigma }\right)}^{4}\right]\text{,} \end{array}$$

where *E* is the expected value and *σ*(=SV) is the standard deviation. Similarly, signal skewness (SK) is the asymmetry of values relative to normal distribution around the mean, hence calculated in the following equation:

(4) $$\begin{array}{c} SK=E\left[{\left(\frac{x\left[n\right]-\mu }{\sigma }\right)}^{3}\right]\text{.} \end{array}$$

Signal slope (SS) is calculated by the following equation:

(5) $$\begin{array}{c} SS=\frac{x\left[n\right]-x\left[n-1\right]}{∆n}\text{,} \end{array}$$

where *x*[*n*] is the xvalue at the current time, and *x*[*n*-1] is the *x* value at the previous time. ∆*n* is the sampling time interval. Moreover, signal area (SA) is obtained by the following integral function expression:

(6) $$\begin{array}{c} SA=\overset{N-1}{\underset{n=0}{\sum }}x\left[n\right]∆n\text{.} \end{array}$$

All statistical features were rescaled between 0 and 1 to normalize the size of the extracted feature vector using the following equation:

(7) $$\begin{array}{c} Z^{\prime }=\frac{Z-\min\left(Z\right)}{\max\left(Z\right)-\min\;\left(Z\right)}\text{,} \end{array}$$

where *Z*′ is the rescaled feature vector and *Z* refers to the original feature vector.

### 2.5. SVM for Detecting UPB Patterns

**Figure 5:**
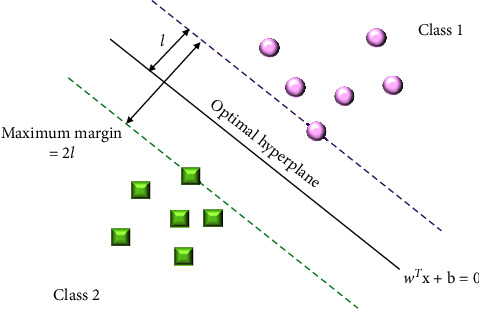
Concept of support vector machine (SVM). The green squares and the pink circles are support vectors. SVM should find the optimal hyperplane (solid black line) divided into Class 1 and Class 2 with a maximum margin (2*l*). Hence, SVM estimates the hyperplane in the two-dimensional space and classifies two classes.

The most popular supervised learning model, SVM, has already demonstrated its excellent performance (i.e., classification accuracy) compared to other classifier models in many studies [39–41]. SVM can explicitly control errors by maximizing margins between two or more classes, known as support vectors [42, 43], as illustrated in Figure 5. The green squares and the pink circles are support vectors. Thus, SVM estimates the optimal hyperplane with the maximum margin (2*l*) between two classes. The optimal hyperplane is defined as follows:

(8) $$\begin{array}{c} d\left(x\right)=W^{T}x+b=0\text{,} \end{array}$$

where *x* represents the input values and becomes *x*=(*x*[0], *x*[1],…,*x*[*N* − 1])^*T*^. *W* refers to the hyperplane's direction as a normal vector of the hyperplane and transposes it to *W*^*T*^.*b* is the position. The optimal hyperplane is determined through *W* and *b*. To calculate *W* and *b*, the margin is defined as the distance between the nearest data points of either class measured perpendicular to the hyperplane. This means maximizing margins while minimizing generalized errors. To reduce errors, we calculated 2*l* by substituting *W* vectors obtained from (8) into (9). Ultimately, we can calculate an optimized hyperplane that maximizes margins between support vectors, which is important to determine the classification accuracy of SVM as follows:

(9) $$\begin{array}{c} Maximum\;margin\left(2l\right)=\max_{W,b}\frac{2}{{\left|\left|W\right|\right|}_{2}}\text{,} \end{array}$$

where $\left\|{\left.W\right\|}_{2}=\sqrt{W{\left[0\right]}^{2}+W{\left[1\right]}^{2}+\ldots +W{\left[N-1\right]}^{2}}\text{.}\right.$ The SVM model was assessed by 10-fold cross-validation to avoid overfitting known as learning biases caused by the classifier's excessive dependency on training data. The training dataset is split into 10-folds containing an equal number of the training dataset. We divided them into the ratio of 8 train sets and 2 test sets for the cross-validation and then tested them 30 times to estimate the variability of the classification accuracies. We subsequently calculated the mean classification accuracy and a standard error of the mean (SEM).

However, this model can lead to an imbalance problem where one of the two classes has more data than the other classes [44]. Thus, we evaluated the reliability of the classification results in the following section to determine whether the classifier results were affected by the imbalance problem.

### 2.6. Reliability of Calculated Classification Results Using AUC

**Figure 6:**
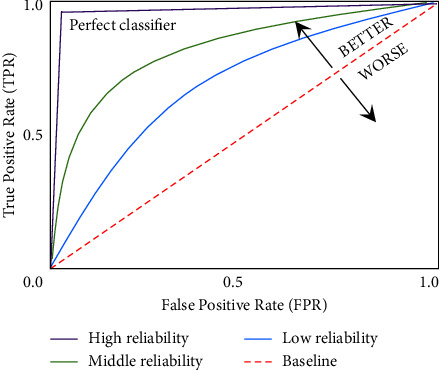
Typical ROC curves and their AUCs. The ROC curves show the relationship between true-positive rate (TPR) and false-positive rate (FPR) to validate the reliability of results calculated by the SVM classifier. TPR is the ratio of correctly judging the UPB elicited by BOGOF as the UPB. In contrast, FPR is the ratio of incorrectly judging the non-UPB as UPB. Through these TPRs and FPRs, the AUC can be calculated by quantifying the entire 2-D region under the ROC curve. The red dotted diagonal line (AUC = 0.5) depicts a baseline. The AUC of the purple line (AUC = 1.0) is considered to be the best reliability in the tested classifier results.

AUC is primarily used to validate the reliability of the results classified by the SVM [45–47].  Figure 6 depicts the typical receiver operating characteristic (ROC) curves and their AUCs which include a true-positive rate (TPR) and false-positive rate (FPR). These statistical indexes such as TPR and FPR are essential for interpreting the reliability of the calculated classification results. AUC estimates the whole two-dimensional area underneath the whole ROC curve (i.e., a kind of integral calculation) from (0,0) to (1,1). Hence, AUC is the range from 0 to 1, and the classification results are the most reliable with an AUC value of 1. The reliability of the results calculated by the classifiers is better as the FPR is lower and TPR is higher. In other words, the closer the AUC is to 1, the better the reliability of the results. If the AUC area is less than 0.5, the calculated classification results are not reliable. After all, it is important to find the largest AUC (close to 1). To quantify the AUC value, we first calculated the TPR and FPR using equations (10) and (11).

(10) $$\begin{array}{c} TPR=\frac{TP}{TP+FN}\text{,} \end{array}$$

where TP refers to the parameter in which the UPB is correctly classified as UPB, and FN indicates that the classifier incorrectly classified UPBs as non-UPBs. Thus, the TPR is the ratio of correctly judging the UPBs evoked by BOGOF as UPBs. In contrast, FPR is the ratio of incorrectly judging the non-UPB as the UPB.

(11) $$\begin{array}{c} FPR=\frac{FP}{FP+TN}\text{,} \end{array}$$

where TN represents that the classifier correctly classified non-UPBs as non-UPBs. FP means that non-UPB is misclassified as UPB.

### 2.7. Detection of UPB and Non-UPB

After completing the SVM classification process, MATLAB® App Designer, a fully integrated development environment, was used to represent UPB classification results by SVM on the computer screen. It is divided into two messages based on the classification accuracy between UPBs and non-UPBs. If the classification accuracy exceeds 80%, the message appears that it is ready to detect UPB patterns; otherwise, the message appears that it cannot detect UPB patterns.

### 2.8. Self-Reported Measurement

In this study, self-reported measurement was used to determine whether impulsivity increased in the BOGOF condition as perceived by the subjects. The subjects answered whether impulsivity was induced in each of the purchase situations of the two tasks. The subjects perceived that impulsivity was induced when BOGOF was present (compared to the absence), suggesting that the experiment was well designed.

### 2.9. Statistical Analyses

All statistical analyses were conducted using the Statistical Package for the Social Sciences, version 25.0. (SPSS Inc., Chicago, IL). Variables were calculated such as normality, means (*μ*), standard deviation (*σ*), and standard error of the mean (SEM) for each task. We used an independent sample *t*-test [48] to compare the difference in the number of clothes purchased by the subjects between Task 1 and Task 2, statistically.

### 2.10. Behavioral Analyses

People who overspend are more likely to make unplanned purchases [49]. Thus, we concentrated on the number of clothes that the subjects intended to buy to investigate the subject's UPB caused by BOGOF condition. The unplanned purchase ratio was calculated using the average and the sum of the clothing purchased by each subject. Thereafter, the independent sample *t*-test is used to investigate a statistical difference in the number of clothing purchased by the subjects.

## 3. Results

### 3.1. Self-Reported Results

A self-reported measurement method was used to determine whether there was a difference in the subjects' perceived impulsivity with and without BOGOF. Many researchers use an alternative variable, a compulsive desire to buy, to measure UPBs. Three items were used to measure the subjects' compulsive desire to buy [50], made on a 5-Likert scale.

There was a significant difference in the perceived impulse purchase intention according to the presence or absence of BOGOF. Subjects perceived impulsivity in the BOGOF condition (*μ* = 3.53; *σ* = 0.80; t(64) = 5.375;  ^*∗∗∗*^*p* < 0.001) than in the non-BOGOF condition (*μ* = 2.40; *σ* = 0.90; *N*.*S*.). It also suggests that our experiment tasks are well designed to compare subjects' impulsiveness and nonimpulsiveness.

### 3.2. Behavioral Results

The behavioral results were obtained from solely the number of clothing purchased by the subjects. The average number of the purchased clothes in Task 1 (*μ* ± *σ*: 6.67 ± 2.27) was higher than that in Task 2 (3.36 ± 2.41). An independent sample *t*-test shows statistically significant differences in the number of clothes purchased between the two tasks (*t*=5.649,  ^*∗∗∗*^*p* < .001), indicating that there is a difference in the UPB pattern between the two tasks. Specifically, in Task 1, the purchased clothes have the sum and standard deviation as follows: knitwear (43 ± 0.60), coat (43 ± 0.70), vest (45 ± 0.66), pants (51 ± 0.76), and suit (38 ± 0.52). Task 2 includes knitwear (31 ± 0.57), coat (19 ± 0.66), vest (25 ± 0.65), pants (24 ± 0.90), and suit (12 ± 0.49). Hence, the difference in the total number of clothes purchased between the tasks were knitwear (*t*=2.472,  ^*∗*^*p* < 0.05), coat (*t*=4.386,  ^*∗∗∗*^*p* < 0.001), vest (*t*=3.742,  ^*∗∗∗*^*p* < 0.001), pants (*t*=3.890,  ^*∗∗∗*^*p* < 0.001), and suit *t*=6.425,  ^*∗∗∗*^*p* < 0.001). This means that the total number in each clothing group can be revealed between the two tasks.

### 3.3. Analyses of Brain HbO Activity in PFC

**Figure 7:**
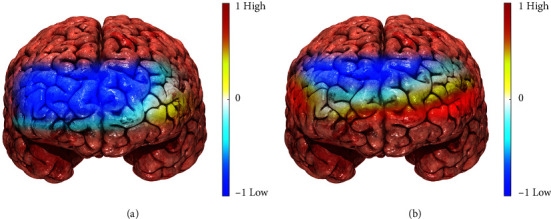
Topographical maps of averaged HbO activities under (a) Task 1 and (b) Task 2. Most areas show little activation except for the left VLPFC in (a). On the other hand, extensive brain activations appear in mPFC, VLPFC, and OFC in (b). These regions have the function of inhibiting UPB as an important predictor of impulsiveness. Thus, these findings provide empirical evidence that the BOGOF condition sufficiently encourages UPB to differentiate it from non-UPB.

We investigated the differences in the presence or absence of UPBs in connection to brain activity.  Figure 7 depicts the topographical maps of averaged HbO activities across all subjects in the PFC areas, and Figures 7(a) and 7(b) correspond to Task 1 (including BOGOF) and Task 2 (excluding BOGOF), respectively.

Except for the left VLPFC, brain activation hardly occurred in Figure 7(a). In contrast, Figure 7(b) indicates the significant brain activations in several regions such as OFC, mPFC, and VLPFC regions, which showed particularly strong activations in the OFC area. Although the DLPFC showed little activation, significant brain activations occurred in the OFC, mPFC, and VLPFC areas, which are known to inhibit impulsivity. As a result, we revealed that Task 2 allows for reasonable consumption as opposed to Task 1.

### 3.4. Classification Results between UPB and Non-UPB Using SVM

**Figure 8:**
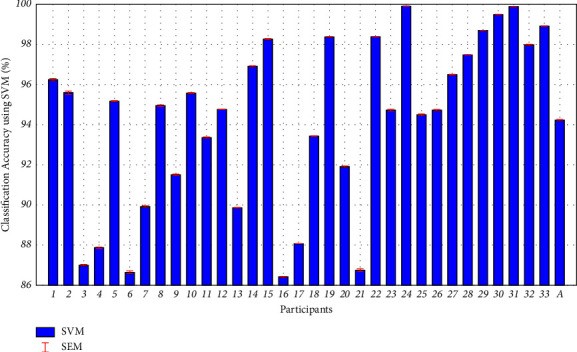
Classification results between UPB and non-UPB using SVM for each subject during cognitive tasks in accordance with the BOGOF condition. The red error bars refer to the standard error of mean (SEM). “A” in the *x*-axis indicates the average classification accuracy across 33 subjects with average accuracy and SEM (94.23%±0.73). The accuracies of all subjects are ranging from 86.42 to 99.90%, suggesting that the fNIRS data could be used as a biomarker to differentiate between UPB and non-UPB.

We used SVM to calculate the accuracies for binary classification between Task 1, which elicits UPBs by BOGOF, and Task 2, which serves as a control task.  Figure 8 exhibits the classification accuracies between UPB and non-UPB using SVM for each subject during cognitive tasks in accordance with the BOGOF. Especially, “A” on the *x*-axis represents the overall average classification accuracy of 94.23% for 33 subjects. The error bars represent SEMs, and the average error bar of “A” is 0.03. All subjects accounted for higher than 86% classification accuracy, which ranged from 86.42% ± 0.02 (accuracy (%) ± SEM) to (99.90% ± 0.01). These provide empirical evidence for differentiating UPBs from non-UPBs.

### 3.5. Reliability Verification of Classified Results Using AUC

**Figure 9:**
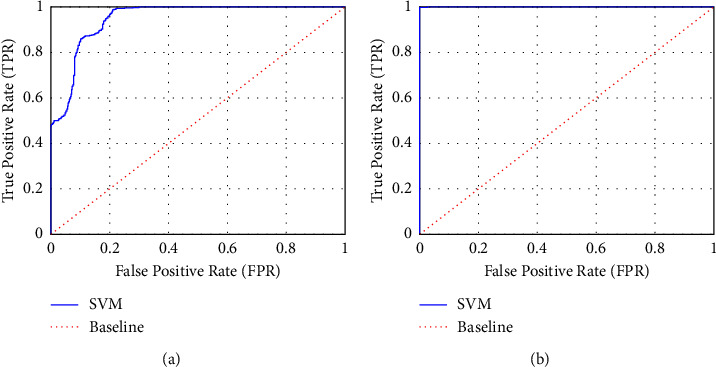
ROC curves and their AUCs of two representative subjects with (a) the lowest accuracy and (b) the highest accuracy. The red dotted diagonals represent the baseline. The reliability of the classification results is not guaranteed if the SVM curves (blue lines) locate below the baseline, but the classification results are reliable if the SVM curves locate above the baseline. Therefore, all SVM results are trustworthy, and (b) is more reliable than (a).

AUC is used to determine the reliability of the classification results, which gives us an intuitive view of the entire spectrum of FPR (*x*-axis) and TPR (*y*-axis).  Table 1 presents the AUC values of all subjects who participated in this experiment. The averaged AUC value is 0.97 across all subjects. Moreover, their AUCs lie between 0.85 and 1.00, indicating that the SVM model is trained perfectly, and their results are highly reliable. More specifically, Figure 9 illustrated the ROCs and their AUCs of two representative subjects with the lowest and highest AUCs among all subjects.  Figure 9(a) refers to the subject's ROC and AUC (0.85) with the lowest accuracy value of 86.42%, and Figure 9(b) illustrated the subject's ROC and AUC (1.00) with the highest accuracy value of 99.90%. As a result, the curves are located above the baseline in both subjects, and their AUC values fully guarantee the reliability of the UPB detection results. In simple words, the larger the AUC value the higher is the reliability of the SVM results, in which both the UPBs and non-UPBs are trustworthily separable. Thus, our study denotes that the SVM model provides high accuracies which are reliable.

### 3.6. Detection Results of UPB and Non-UPB Patterns

**Figure 10:**
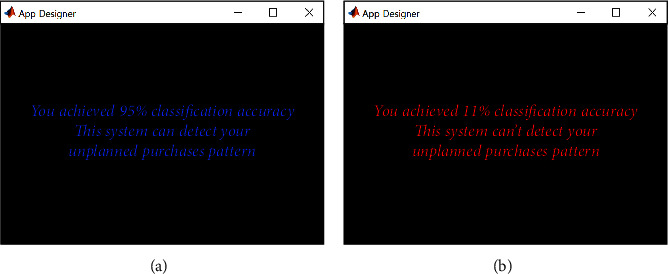
Screenshots of the detection results for UPB patterns. If the classification accuracy is above 80%, a message indicates that you are ready to detect unplanned purchase patterns (a); otherwise, indicates that you are not able to detect unplanned purchase patterns (b). In this experiment, all subjects received a message stating that this system can detect UPB patterns because each subject achieved a classification accuracy of higher than 86%.

Figure 10 illustrates the screenshots of the detection results for UPB patterns. All subjects received a message that this system can detect UPB patterns because each subject had reached a classification accuracy of more than 86% in this experiment.

## 4. Discussion

### 4.1. Proposed UPB Identification Methodology with BCI and Self-Reported Measurement

Research related to promotion strategies focuses on detecting and predicting people's UPB patterns [1]. However, it is difficult to measure the actual UPB in the lap setting. Therefore, UPB has so far relied on qualitative and quantitative research such as interviews and surveys. In line with this trend, this study also confirmed that a compulsive desire to buy increased during BOGOF using self-report measurement. It also demonstrates that our experiments are well designed. Several studies, however, emphasize that these tools still must be used with caution because they are influenced by the subjects' perceptions and memories [8]. To supplement the shortcomings of traditional marketing research methods, we present the UPB identification methodology to classify between a UPB and a non-UPB as illustrated in Figures 7 and 8. Eventually, we can identify the UPBs through a machine learning-based classification approach using fNIRS-SVM along with the self-reported results.

### 4.2. High Classification Accuracy in the Proposed Methodology Compared to the Existing Research Methodology

In our study, we proposed the optimal measurement methodology based on fNIRS-SVM, which would aid and improve the correct identification of UPBs caused by BOGOF. The proposed method along with self-reported measurements can serve as an optimal measurement tool to detect unplanned and impulsive purchase patterns. Likewise, in previous studies, measurement tools for impulsive detection have also existed, including clinical and neuropsychiatric tests. According to a previous study [51], psychometrical questionnaires such as Barrat's impulsiveness scale version 11 and the International Personality Disorder Evaluation Screening Questionnaire were used to detect people's impulsivity. These results are consistent with the SVM results by obtaining the impulsivity classification accuracy above 76%. Another study has reported the potential of the fNIRS-SVM classification approach between impulsive and nonimpulsive adolescents, which achieves a classification accuracy greater than 90%. This result was identical to the clinical assessment results by showing a significant difference in scores between the two adolescent groups [52]. Similarly, we achieved an average accuracy of 94% by detecting UPBs across 33 people, which was higher than the results of the previous study [52] as illustrated in Figure 8. Our study shows the highest achievement among previous achievements for detecting UPBs.

### 4.3. Detection of Low Brain Activity in the UPB

Many studies have reported that the more impulsive buying tendency people have, the lower is the brain activity in their PFC. For example, typical symptoms related to impulsiveness include obesity and binge-eating disorder [53]. They found that the obesity group had a lower fNIRS-based PFC response than the normal-weight group, indicating a connection between impulsiveness and a specific obesity phenotype. Another study found that the control group had higher prefrontal activation than ADHD children with high impulsiveness [54, 55]. Similarly, our study illustrates that Figure 7 depicts low brain activation at the PFC as a result of BOGOF. We have also confirmed that BOGOF elicits UPBs from the subjects' self-reported results. Thus, we revealed low brain activity because of UPBs at the PFC for the first time.

## 5. Conclusions

We proposed the optimal measurement methodology applied with fNIRS-SVM that can classify UBP patterns caused by BOGOF tasks and non-UBPs caused by control tasks and then validate their excellent classification results by AUC. As a result, we achieved an average accuracy above 94% by utilizing patterns of promotion strategy for UBPs. The classification result's reliability is validated by satisfying the AUC values above 0.97. We also found that the brain activity for UPBs was lower during the BOGOF tasks than during the control tasks at the PFC. This is consistent with the self-reported results that the subjects perceived an increase in impulsivity when they were exposed to BOGOF. Therefore, this study raises awareness of consumers' UPB and shows the possibility of applying optimized UPB measurement methodology to various applications such as mobile and PC in terms of computer-aided detection.

## Figures and Tables

**Table 1:** AUC results across 33 subjects.

Subjects	AUC	Subjects	AUC	Subjects	AUC
Subject 1	0.99	Subject 13	0.95	Subject 25	0.98
Subject 2	0.96	Subject 14	0.99	Subject 26	0.97
Subject 3	0.92	Subject 15	0.99	Subject 27	0.98
Subject 4	0.95	Subject 16	0.95	Subject 28	0.97
Subject 5	1.00	Subject 17	0.85	Subject 29	1.00
Subject 6	0.93	Subject 18	0.99	Subject 30	1.00
Subject 7	0.96	Subject 19	1.00	Subject 31	1.00
Subject 8	0.94	Subject 20	0.97	Subject 32	0.99
Subject 9	0.96	Subject 21	0.97	Subject 33	1.00
Subject 10	0.99	Subject 22	1.00	Average	0.97
Subject 11	0.99	Subject 23	0.99	SD	0.03
Subject 12	0.97	Subject 24	1.00	SEM	0.01

## Data Availability

The raw data used in the study are available from the corresponding author upon reasonable request.
